# Correlation between disability and MRI findings in lumbar spinal stenosis

**DOI:** 10.3109/17453674.2011.627496

**Published:** 2011-11-24

**Authors:** Andrew J. Haig

**Affiliations:** Physical Medicine and Rehabilitation, The University of Michigan, Ann Arbor, Michigan, USA

Sir—The work of Sigmundsson and colleagues (2011) represents a very good analysis of a cohort of persons subjected to surgery for spinal stenosis. However, the discussion of the disease itself far over-reaches any conclusion that can be drawn from this highly selected population that contains no control group. The Michigan Spinal Stenosis Study, a controlled study including asymptomatic persons and persons with back pain, addresses the same issues with contradictory findings. We found that, of 14 different measures of the lumbar vertebrae, including the thecal sac area and ‘smallest two area’ measures similar to those used in their study, only two measures (spinal canal diameter and smallest two spinal canal diameters) had any relationship to the clinical diagnosis of stenosis, and these two had no discriminant value (Haig et al. 2007). Furthermore, these measures have no relationship to level of disability including three standardized scales, a 15 minute laboratory walking test, or pedometer-measured community walking at inception (Geisser et al. 2007). Spinal measures did not predict function or pain at follow-up more than 18 months later (Haig 2006). The study included primarily people who were not referred for surgery. However, other large cohorts that do include asymptomatic persons fail to show any important relationship between the imaging and clinical presentation of stenosis (Haig and Tomkins 2010). We must disagree with the authors' opening statement that “MRI is the modality of choice when diagnosing spinal stenosis'. Evidence to date tells us that the test only rules out dangerous diseases and assists with surgical planning.

The decision of a surgeon to operate and of a patient to accept an operation is a complex one that goes beyond objectivity on the part of both parties (Lurie et al. 2008, Deyo 2009). Any conclusion about the disease itself—including a belief that smaller canals relate to worse symptoms-but also pathophysiological observations that women have more multilevel findings, the percentage of persons with spondylolysthesis, etc.—must be viewed in light of the surgeon and patient's own belief systems and biases.

Clinical spinal stenosis remains a complex and poorly defined, and poorly validated syndrome. There may well be some relationship between spinal measures and disability. Despite the well intentioned work of Sigmundsson et al. modern research is showing that a surgical convenience sample does little to clarify the syndrome.

## References

[CIT0001] Deyo RA (2009). Imaging Idolatry. Arch Intern Med.

[CIT0002] Geisser ME, Haig AJ, Tong HC, Yamakawa KS, Quint D, Hoff JT, Miner JA, Phalke VV (2007). Spinal canal size and clinical symptoms among persons diagnosed with lumbar spinal stenosis. Clin J Pain.

[CIT0003] Haig J, Tomkins C (2010). Diagnosis and treatment of lumbar spinal stenosis. JAMA.

[CIT0004] Haig AJ, Tong HC, Yamakawa KS, Parres C, Quint DJ, Chiodo A, Miner JA, Phalke VC, Hoff JT, Geisser ME (2006). Predictors of pain and function in persons with spinal stenosis, low back pain, and no back pain. Spine.

[CIT0005] Haig AJ, Geisser ME, Tong HC, Yamakawa KS, Quint DJ, Hoff JT, Chiodo A, Miner JA, Phalke VV (2007). Electromyographic and magnetic resonance imaging to predict lumbar stenosis, low-back pain, and no back symptoms. J Bone Joint Surg (Am).

[CIT0006] Lurie JD, Berven SH, Gibson-Chambers J, Tosteson T, Tosteson A, Hu SS, Weinstein JN (2008). Patient preferences and expectations for care: determinants in patients with lumbar intervertebral disc herniation. Spine.

[CIT0007] Sigmundsson FG, Kang XP, Jönsson B, Strömqvist B (2011). Correlation between disability and MRI findings in lumbar spinal stenosis. A prospective study of 109 patients operated on by decompression. Acta Orthop.

