# Supplementary material for: Correlation between disability and MRI findings in lumbar spinal stenosis
Source: Acta Orthop. 2011 Nov 24;82(5):637–8. doi: 10.3109/17453674.2011.627496 (PMC3242966; doi:10.3109/17453674.2011.627496)
Supplement: Related texts

# Letter: Correlation between disability and MRI findings in lumbar spinal stenosis

**Published:** 2011-11-24

**Authors:** Freyr Gauti Sigmundsson, Björn Strömqvist, Bo Jönsson

**Affiliations:** Ortopediska Kliniken, BlekingesjukhusetKarlshamn; Department of OrthopedicsUniversity Hospital of Skåne, Lund, Sweden

*Sir—*We appreciate Dr Haig's interest in our study (Sigmundsson et al. 2011) and also recognize the important works having been performed by him and his collaborators (Haig et al. 2007, Haig and Tomkins 2010).

It goes without saying that a decision to operate, which should be a contract between the surgeon and the patient, is biased and certainly influenced by beliefs and previous experiences.

However, the clinical picture of spinal stenosis with pseudoclaudication symptoms, i.e. radiating symptoms that prevent walking for long distances and is relieved by flexion of the spine, is fairly distinct, and this is the type of patients being selected for surgery, where MRI is used for confirmation of the diagnosis.

We agree that a general correlation between measurement of dural sac area and clinical symptoms is most unreliable. Asymptomatic spinal stenosis is not unfrequent but this fact is also true for example lumbar disc herniation, hip osteoarthritis, meniscal injury and so forth. What our study mainly discusses is whether positive and negative prognostic factors can be identified in patients with clinical symptoms enough to justify surgery. In this group there is a relationship between spinal measures and disability in some aspects as presented in our study, and these facts can be used in the clinical situation when surgery is discussed with a symptomatic patient. This would be of clinical significance as satisfaction with surgery is only obtained in 65–80 % of patients. We draw no further conclusions from our study, which, however, in our opinion is another small piece in the puzzle of “complex and poorly defined and poorly validated syndrome” called lumbar spinal stenosis.

## References

[CIT0001] Deyo RA (2009). Imaging Idolatry. Arch Intern Med.

[CIT0002] Geisser ME, Haig AJ, Tong HC, Yamakawa KS, Quint D, Hoff JT, Miner JA, Phalke VV (2007). Spinal canal size and clinical symptoms among persons diagnosed with lumbar spinal stenosis. Clin J Pain.

[CIT0003] Haig J, Tomkins C (2010). Diagnosis and treatment of lumbar spinal stenosis. JAMA.

[CIT0004] Haig AJ, Tong HC, Yamakawa KS, Parres C, Quint DJ, Chiodo A, Miner JA, Phalke VC, Hoff JT, Geisser ME (2006). Predictors of pain and function in persons with spinal stenosis, low back pain, and no back pain. Spine.

[CIT0005] Haig AJ, Geisser ME, Tong HC, Yamakawa KS, Quint DJ, Hoff JT, Chiodo A, Miner JA, Phalke VV (2007). Electromyographic and magnetic resonance imaging to predict lumbar stenosis, low-back pain, and no back symptoms. J Bone Joint Surg (Am).

[CIT0006] Lurie JD, Berven SH, Gibson-Chambers J, Tosteson T, Tosteson A, Hu SS, Weinstein JN (2008). Patient preferences and expectations for care: determinants in patients with lumbar intervertebral disc herniation. Spine.

[CIT0007] Sigmundsson FG, Kang XP, Jönsson B, Strömqvist B (2011). Correlation between disability and MRI findings in lumbar spinal stenosis. A prospective study of 109 patients operated on by decompression. Acta Orthop.
